# Examining customer intentions to purchase intelligent robotic products and services in Taiwan using the theory of planned behaviour

**DOI:** 10.1186/s40359-024-01683-z

**Published:** 2024-06-15

**Authors:** Yu-Hung Tai, Precious Toby T. Nwachukwu, Ben A. LePage, Wei-Ta Fang

**Affiliations:** 1 London Graduate School, University of Chichester, Chichester, West Sussex PO19 6PE UK; 2 Douglas Business School, Tsim Sha Tsui, Hong Kong, P.R. China; 3 Department of Educational Management and Policy Studies, University of Pretoria, Pretoria, South Africa; 4 Graduate Institute of Sustainability Management and Environmental Education, College of Science, National Taiwan Normal University, Taipei, Taiwan; 5 Academy of Natural Sciences, 1900 Benjamin Franklin Parkway, Philadelphia, PA 19103 USA

**Keywords:** Product, Semiconctor, Taiwan, Theory of Planned Behavior

## Abstract

**Background:**

The literature for assessing online and offline shopping behaviours that are linked to intelligent robotic goods and services is inadequate. In this study, we applied the Theory of Planned Behaviour model for guidance regarding how consumer behaviour affects their purchase intentions for intelligent robotic goods and services.

**Methods:**

Data from 408 respondents were gathered through an online questionnaire binned into Online and Overall Shoppers, and analysed using SPSS, AMOS, and Covariance-Based Structural Equation Modelling software to evaluate the appropriateness of the measurements and to confirm data reliability, convergence, divergence, and validity. These tools were also used to track and test hypothesized relationships between the variables and model constructs used in this study.

**Results and conclusions:**

The overarching outcomes from the data analyses indicated the Ease of Usage, Brand Perception, and Product Pricing variables causally impacted the TPB model constructs, namely Attitude, Subjective Norms, and Perceived Behaviour Control for the two populations tested with respect to their intention to purchase intelligent robotic goods and services. The reliability measurements for Ease of Usage, Brand Perception, and Product Pricing are discussed. The results are important for companies and future investors because opportunities to study the complex relationships that ultimately drive consumer behaviour and their intention to purchase intelligent robotic goods and services are provided.

## Background

**Fig. 1:**
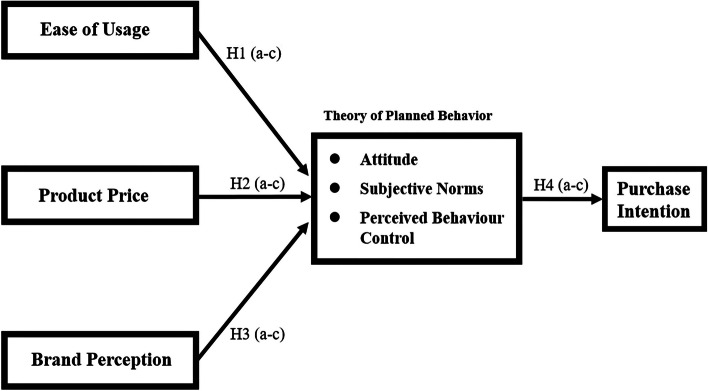
Conceptual model illustrating the ease of usage, product price, and brand perception variables that impact the three TPB constructs (attitude, subjective norms, and perceived behaviour control) that impact purchase intentions of the overall shopper group. the hypotheses that were tested are included. source: Tai [11]

Consumer buying behaviour has been the focus of advertising and marketing studies for decades [1]. Purchasing decisions are based on the type and amount of information received by the consumer [2]. The arrival of the internet and smartphones have substantially changed consumer purchasing behaviours, especially in the last decade [3]. From the standpoint of developing and ultimately selling a product, its logical then to understand what determines or drives consumer buying behaviour. The Theory of Planned Behavior model (TPB) is a model developed by Ajzan [4] that is commonly used to predict human behavior (Fig. 1). It is a tool used to measure a model’s constructs that are comprised of Attitude (A), Subjective Norms (SN), and Perceived Behavior Control (PBC), which then causally influences a person’s intentions and ultimately their behavior [5–7]. Technological advancements and innovations have made the incorporation of Intelligent Robotics (IR) into our lives, a reality [8–10]. However, information on the behaviours of internet (Online) and brick-and-mortar (Offline) shoppers that are linked to IR goods and services is inadequate.

The application of the TPB to the Online and Offline purchasing behaviours of shoppers has been applied across a wide range of disciplines and domains [12–14]. Our intent was to reconnoitre whether emerging automation and robotic solutions, now called IR can meet consumer shopping needs and demands by applying the TPB to computer technology advancement. The emergence of Artificial Intelligence (AI) in this era and the impact of Gordon Moore’s law [15] indicates computing power will double every 18 to 24 months; however, not everyone can fully understand the implications of the speed of this progress [16]. Semiconductor technology has surpassed the rules of Moore’s Law [17] in response to the rapid advancement and growth of new technologies since 1979 and Alphabet’s breakthrough in quantum computing that has a computational state-space of approximately 10^16^ dimensions [18, 19]. These new innovative technologies and advancements are now linked with smart energy networks [20] and have enhanced computerized management, network communication, and intelligent analysis through perception and interconnection. This has enhanced the user’s perceived usefulness of the inter-connectedness and incorporation of intelligent system technologies into our daily lives [21, 22].

As such, the next step in the evolution of information management is to incorporate information technologies with intelligent products, permitting humans to be involved in the discourse and rationalization of decision-making using IR. It’s been iterated that East Asia is at the frontier for promoting robotic technologies [23, 24]. Transitions of this magnitude however, require national-level procedural and behavioural changes. Thurbon and Weiss [23] and others [11, 25–29] examined national-level industrial transformations associated with frontier development projects in Asia and found that the less well-developed countries struggled with predatory rulers or well entrenched rent-seeking elites, that stall out or delay project advancement. Furthermore, the pressure associated with increasing a nation’s economic growth, competition from within and outside the country, improving-maintaining-developing government-business relationships, and the uncertainty of success in frontier development projects, such as IR all determine the level conservatism, involvement, and/or hands-off approach taken by the private and government sectors [23, 25].

Success on the other hand comes with risk and making hard decisions. South Korea is an example that illustrates the government’s role identifying the challenges associated with the evolution of IR, cutting through the red tape, and coordinating and mobilizing the resources needed to deliver socially productive outcomes [23]. The Korean government in 2003 recognized IR as a strategic industry and by 2005 the Ministry of Information and Communication inaugurated the IR test-bed initiative. The Intelligent Robots Development and Distribution Promotion Act was passed in 2018 by the Ministry of Trade, Industry, and Energy, the Ministry responsible for the IR industry that announced the Intelligent Robot Industry Development Acceleration Strategy [23, 30]. In China, the government announced robotics was a new strategic industry and recognized a preliminary plan in 2011 to begin local development in this technology. The Chinese and Japanese governments launched the National Plans for Robotics Industry Development in 2015 [23, 25].

Thurbon [3] suggested that the evolutionary path of Taiwan’s developmental state has been linear and stagnant since the 2000s due to social legitimacy and political leadership. This is however, no longer the case because Taiwan has imbued its financial activism efforts and connected them to their national development productivity development plans and strategies [30]. Taiwan is now a leader developing products such as semiconductors, 5G telecommunications, AI, and the Internet of Things [31]. The Taiwanese (R.O.C.) government started implementing the integration of human and intelligent products in 2022 through the Ministry of Science and Technology, Ministry of Economic Affairs, National Development Plan, and Reform Commission. These and other ministries and commissions were invited to select 100 technology start-up firms to participate in the 2022 Consumer Electronics Show [32]. Of the 100 companies that were identified and selected, 27 were from the smart healthcare sector, 25 were focused on AI and information security, 25 were in the semiconductor, space, and communication applications areas, and 23 were digital technology companies. The Covid-19 threat and subsequent Omicron response forced the venue [32] to be showcased in a virtual and real dual-track format, utilizing a multi-faceted digital technology marketing strategy. This allowed Taiwan to display its new competitiveness in the technological innovation and digital connection space to the world [32]. It then generated international popularity and enticed opportunities for global capital injection. Linking AI goods and services to humanity on a contemporary day-to-day basis requires the delivery of data and interpretation of outcomes quickly, necessitating a collaborative astuteness that identifies patterns and insights that the TPB is geared to provide.

### Intelligent robotics (IR) in Taiwan

Although robots have been used in the manufacturing industry worldwide for decades, including Taiwan, the use of IR in Taiwan is new. As a result, the main author conceived this project in 2022 and the data were collected soon thereafter. The data were analysed and published as a thesis in 2023 [31] and parts of the study are presented herein. Hypotheses that test the impacts of Product Pricing (PP), Brand Perception (BP), and Ease of Usage (EU) on the TPB constructs, which then influence consumer Purchase Intentions (PI) of IR goods and services are the focus of this study. The Offline Shopper group data and impact of other variables on the TPB constructs will be the foci of other papers that are in preparation. The TPB indicates that a person’s choice to be involved in a particular behaviour or logical cognition [33], including AI is endorsed and can be anticipated through their purpose to engage in that behaviour and/or cognition. The three TPB constructs, A, SN, and PBC determine an individual’s intentions and behaviour that are then influenced by its consequences [34, 35]. A conceptual model illustrating the variables that impact the TPB constructs that ultimately influence a person’s Purchase Intent (PI) for IR goods and services or any other goods or services is provided and the hypotheses we tested are provided in Fig. 1. The TPB is built around the premise that a person’s personal preferences, attitude, and support from important others will affect their behavioural intentions [36], while A and SN will directly affect their intentions and indirectly their behaviours [4]. Specifically, Rational Behaviour Theory states that behaviour occurs from the individual's conscious control to maximize benefits and minimize risks and the TPB model can help predict individual decision-making behaviours [37].

### Variables and TPB constructs

Wang et al. [38] indicate AI is being used more effectively in for e-commerce to achieve business goals and milestones because AI can be trained to recognise the relationships between the variables being tested and TPB constructs. This indicates that trust in a service or product is important with respect to the model constructs and a person’s behavioural intention to procure any service or product [38]. Given that AI, especially IR has been growing for decades and its influence infiltrates diverse segments of the economy [39], its impact has had remarkable achievement in the advertising, business, finance, education, and health care fields [40, 41]. The usefulness and/or efficacy of a service or product from the customer’s point of view is their perceived utility [42]. Moreover, EU can attest how a consumer perceives a particular product or its utility [43]. Trust plays a bigger role in determining the customer's perception of EU for any service or product [44]. The concept of value transcends monetary worth. In this case, it’s directly related to the amount of effort needed to learn a new technology for any service or obtain products that ultimately indicates a customer’s willingness to purchase that service or product [45]. The EU can be explained as the willingness in a person’s behaviour to buy and try any product or service that provides them with tangible and/or intangible benefits [46]. Therefore, the EU of IR goods and services was included, rather than the intention to inform the development and response to IR technology in this study. Huang [47] found that perceived EU and the perceived use of health-intelligent products influence consumer Purchase Intentions (PI). Sun et al. [48] argued that facilitating the effects on the A, SN, and PBC constructs impacted the functionality and usability of mobile telephone payment apps on the repurchasing intentions of consumers in China. Rachmawati et al.’s [49] investigation illustrated that perceived benefits, EU, SN, and personal innovation have a direct effect on the A of using social media. Furthermore, Rini et al. [50] showed that the A, PBC, and SN of the online shoppers they measured had significant effects on their PI and behaviour.

The TPB using Product Price (PP) and intention to buy a service or product at a stipulated price is an important construct of consumer choice [51, 52]. For example, what drives the choice of local or seasonal food requires an analysis of the importance of key personal and/or social motives. Product Price denotes the tangible value of the product or the status of the product or service and its ownership following procurement [53]. It’s a significant factor that’s regulated and measured for the consumer’s approval, as well as real or perceived values that are indicative of purchasing a service or product [54, 55]. The market strategy is to create income and distinguish production costs [56]. The importance of PP enhances the positive effect and promotion of a person’s intention to purchase and the cost of the product [55, 57]. Product Price perceptions have relations to perceived value that directly impact consumer purchasing behaviours and intentions [58]. Therefore, the price and quality of a service or product influences the perceived value and risk associated with that service or product, which could affect a person’s PI. Observations indicate that PP positively influences A, which significantly impacts the consequences of a person’s PI [59–61].

The perception of a product’s low-price guarantees price outcomes and customer perceptions of price fairness that are crucial aspects that influence the contentment and PI of a customer [60, 62]. Social Norms and self-congruence were arbitrators between customer PI and the services offered or PP was negatively influenced if the quality of service or pricing was low [63]. Subjective product knowledge has initiated an imperceptible value obligation in customer robotics-advisory adoption in the banking industry [64]. Subjective product knowledge moderates the relationship between perceived PI and PBC and plays crucial roles equally in the social comparison information and the group’s influence, and their own perceived control [60]. It’s also a moderating variable for the relationship between A and PI [64].

Brand perception (BP) is what consumers think or feel about your goods or services [65]. The human characteristics that are associated with a brand is a construct referred to as brand personality [66]. Brand perception and personality are closely related because they allow people to express their self, ideal self, or a dimension of their self by being associated with a brand [66]. The relationship a consumer forms with a particular brand creates loyalty to that brand, distinguishing it from other brands/competitors [67].

### Theory of planned behaviour (TPB) applications for intelligent robotics (IR)

The use of the TPB has been widespread and recently applied in several domains including behaviour, health, education, and the environment [68, 69]. Most studies are from China, Malaysia, United Kingdom, and the United States and focus on environmental issues and sustainability [68]. However, publications on the TPB application for IR that concentrate on fashioning machines that can execute jobs that necessitate physical interaction with the environment and people are scarce [11]. Recent studies are based on student perceptions and motivations to study AI, most of which are centred on AI eHealth technologies [70]. Many are focused on natural language processing and machine learning [71], whereas, Thayyib et al. [72] admitted that most scholars were focused on AI machine deep learning in business and management.

With the innovation in engineering technology, automation, and information technology, the progress of robotics has been elevated from the hardware to application program level and can effectively perform repetitive tasks that humans perform [73]. Likewise, the value of computerization has become part of the development of processes and procedures in the service industry, performing unconventional tasks from previously standardized operations performed by robotics in the manufacturing industries [74]. The primary objective for using robots was to supplant human labour, increase efficiency, and decrease labour costs, all of which saved money [74]. Service robotics are now being widely used in diverse business ecosystems and their classification includes tangible and intangible services that engage people or objects when they are connected and embedded into larger knowledge- or cloud-based systems when providing tangible services [74–76]. The basic principles of automation in service robotics are used or substituted for uncomplicated labour-intensive tasks [75], and workflow is allocated into automated and non-automated responsibilities. Intelligent robotics will ultimately require the physical competencies humans possess, such as the tactile senses we feel with our fingers for labour-intensive agility or precision [77]. Robots endowed with physical or humanoid appearances are being manufactured based on consumer product preferences and cost considerations and are considered the intangible services [75]. The anthropomorphic characteristics of robotics can motivate shoppers to develop a feeling of trust for a product, cultivate PI, and discover contentment in the use of the product [78]. Intelligent Robots are not prone to mental stress or burnout and new tasks can be performed effortlessly [79].

The assumption is that in the future, automation using machines and computers without the assistance of humans and the 5th Industrial Revolution (Industry 5.0) will be based on intelligent automation [80]. This will require employing and integrating an Artificial Intelligence of Things platform, Big Data, Cloud Computing, and IR, with efficient and active management systems, including cognitive computing [9, 80]. Starting in 2020, Industry 5.0 will lead the upgrade and transformation of domestic industries using AI [81]. The perceived ease of usage is an important factor of information technology studies, with the acceptance of the Internet and World Wide Web with no exemption being significant [82]. This has been observed in studies that focused on the perceived ease of usage of technologies in an operational and/or marketable setting in the industrialized nations [82, 83].

### Hypotheses tested

As noted previously, most of the IR research has been associated with projects that are large to technically complex. Said another way, IR hasn’t been part of our social fabric where we are knowingly interacting or replacing day-to-day tasks with a machine to simplify our daily lives (e.g., vacuum cleaner). Therefore, we are short on studies that examine consumer IR perceptions and behaviours, the social connectivity of humans to robots, and even children’s attitudes towards AI robots as teachers [11, 84–86]. To fill this gap, we examined consumer PI for IR goods and services quantitatively, using PP, BP, and EU variables together with the TPB constructs. The objectives were to investigate the impacts of consumer EU, PP, and BP on the TPB constructs and their subsequent impact on the PI of IR goods and services. The sample population was comprised of 408 respondents [11]. To keep the size of the paper manageable, the original population and accompanying data were divided into the Overall (total population) Shopper (H) group and the Online (ß) Shopper group, which is a subset of the Overall Shopper group. Contributions that are currently being prepared, will follow the same procedures and use the same tests to examine the relationships between 1) the Offline (Ώ)-Overall (H) Shopper groups and 2) the Online (ß)-Offline (Ώ) Shopper groups. The following hypotheses were constructed and tested to aid in our investigation (Fig. 1):

- Hypothesis 1: Ease of Usage (EU) is positively related to Attitude (A; H1a, β1a), Subjective Norms (SN; H1b, β1b), and Perceived Behaviour Control (PBC; H1c, β1c);
- Hypothesis 2: Product Price (PP) is positively related to Attitude (A; H2a, β2a), Subjective Norms (SN; H2b, β2b), and Perceived Behaviour Control (PBC; H3c, β3c);
- Hypothesis 3: Brand Perception (BP) is positively related to Attitude (A; H3a, β3a), Subjective Norms (SN; H3b, β3b), and Perceived Behaviour Control (PBC; H3c, β3c); and.
- Hypothesis 4: Attitude (A; H4a, β4a), Subjective Norms (SN; H4b, β4b), and Perceived Behaviour Control (PBC; H4c, β4c) positively influence Purchase Intentions (PI).

## Methods

Tai [11] utilized a cross-sectional quantitative research design to examine consumer behavioural responses associated with the purchase of IR goods and services using the TPB constructs, three consumer variables, and the questionnaire assumptions, design, and responses to validate the aforementioned hypotheses [87, 88]. These data were then analysed using a Covariance-Based (CB)-SEM approach. As noted by Bowen and Guo [1] SEM is a general or umbrella statistical model within which other statistical analyses such as analysis of variance (ANOVA), analysis of covariance (ANCOVA), multiple regression, factor analysis, path analysis econometric models of simultaneous equation and non-recursive modelling, multilevel modelling, and latent growth curve modelling are nested [1]. Structural Equation Modeling is generally used to explain the consortium of statistical analyses used to measure relationships simultaneously through visualization and model validation processes [1, 89]. Confirmatory Factor Analysis (CFA) helps assess the variables and relationships in a model that then allow hypotheses to be supported or rejected [89–94]. Models using these types of analyses can be combined with simultaneous econometric equations to examine the complex interactions between the dependant and independent variables [95, 96]. Simultaneous econometric equations were used in this study to address the relationships between the dependent variables.

This methodology has been applied to hundreds of medical, sociological, environmental, and marketing studies that use non-probabilistic convenience sampling surveys such as questionnaires to analyse the responses from different populations or groups [1, 97, 98]. We explored whether IR could meet the needs of the public using the TPB using the responses from the two populations. It became evident that insight on the trends and/or data on consumer behaviour that companies will ultimately need to identify their target audience and then develop and implement marketing and sales strategies for IR purchases were nested in the results. SPSS (v. 19.0) and AMOS (v. 19.0) were used to analyse the reliability and validity of the data. Examination of the similarities and differences between the populations using CB-SEM to test hypotheses, interpret results, and provide conclusions is an industry/research standard [99–101].

Scholars have argued and observed that if a researcher aims to scrutinize the cause-and-effect relationship(s) between independent and dependent variables, SEM analysis is then the desired statistical tool [100]. It is argued that SEM is an attractive multivariate statistical approach that has the ability to test the causal relationships between constructs with multiple measurement items [97, 100, 102]. Structural Equation Modelling can be performed using CB-SEM or variance-based (VB-SEM) approaches. The CB-SEM is used for data sets possessing a large sample size (population) that is evenly distributed, the cause-and-effect relationship is appropriately stated, and the relationships are confirmed or rejected through hypothesis testing [99, 102]. The CB-SEM analysis procedures include the Maximum Likelihood (ML) estimation technique [97, 102]. The VB-SEM approach is more appropriate for exploring relationships among and between constructs and is more robust concerning the assumption of normality and sample size [99, 100]. It is used when cause-and-effect relationships cannot be ensured [99, 100]. The VB-SEM approach utilizes the Ordinary Least Square (OLS) regression-based estimation technique [97, 99, 103]. Both methods are similar in measuring the validity of the variables and convergent and discriminant validity for retrieving the goodness or the validity of the items to be measured [97, 99, 100, 103].

In the cross-service industry field where independent, but inter-connected business sectors work collaboratively to better meet consumer needs, the increased use of service and social robotics in the future can be assumed based on its current significance and use in the marketplace. AMOS software was used because it utilizes ML estimation techniques, supporting our choice to use the CB-SEM approach to analyse the data [102, 104]. The selection and use of the CB-SEM approach is also appropriate for theory testing and confirmation because it requires a large sample size, data are uninterrupted in a reflective mode, and normality assumptions must be considered [97, 99, 105]. This study possessed all of these characteristics.

### Participants and procedure

The questionnaire for this study was distributed electronically and made available online from October 25 to 30, 2022, and a total of 408 responses were received. None were invalid. The responses that were returned were equally divided with 204 Online and 204 Offline shoppers (total # of respondents = 408). The questionnaire data provided insight into human opinions and reactions related to IR and the hypotheses of this study because the rise of IR has and will continue to replace people’s jobs [99, 100, 105] and many professionals and workers will face extraordinary challenges [74]. To better manage the size of the study and amount of analytical data that would be generated, only the relationships between the Online and Overall Shopper groups are being considered at this time. Analyses of the relationships between 1) the Offline and Overall Shopper groups, 2) Online, Offline, and Overall Shopper groups, 3) Online and Offline Shopper groups and 4) the effects of additional variables and demographics on the TPB constructs and subsequent impact on consumer PI for IR goods and services will be the foci of future contributions. The questions that were used in the questionnaire for this study are from Tai [11] and provided in Table 1.

**Table 1:** Questionnaire items used to measure the relationships between the TPB constructs

**Variables and TPB Constructs**	**Questions**	**Sources**
**Ease** **of Usage (EU)**	1. I can easily search and download public data from IR 2. I can easily become skilled using IR 3. I can clearly understand every menu and feature available in IR 4. IR makes my search and purchase for life\s needs more efficient 5. IR increases my productivity at work 6. I can perform my needs using IR even if there is no one around to help me 7. I can perform my needs using IR using only a simple manual or online help for reference	[83, 106–110]
**Product** **Price (PP)**	1. I think the price is an important factor in evaluating the quality of IR 2. I don’t care about the price of IR when I decide to buy IR 3. I chose a higher-priced IR with the same features	[111, 112]
**Brand** **Perception (BP)**	1. Foreign brands are of good quality 2. Well-known brands are of good quality 3. Unknown brands are of poor quality 4. I usually buy the best-selling brands 5. I prefer buying well-known brands 6. Brands influence my choice of purchase 7. Brands make it easier to choose the product	[113]
**Attitude (A)**	I think buying IR to be … 1. Bad <—-—-—> Good 2. Useless <—-—-—> Useful 3. Unpleasant <—-—-—> Pleasant 4. Unimportant <—-—-—> Important 5. Harmful <—-—-—> Beneficial	[114]
**Subjective** **Norms (SN)**	IR Issues: 1. My colleagues or friends are buying IR products 2. Someone who has a significant influence on me (such as my boss or teacher) thinks I should be buying IR products 3. People who are important to me (such as my relatives or family members) support me in purchasing IR products 4. I can decide for myself whether to buy IR products	[115, 116]
**Perceived** **Behaviour** **Control (PBC)**	IR Issues: 1. I can make my decision to buy IR products independently 2. I have the financial capability to buy IR products 3. I have complete information and awareness regarding where to buy IR products 4. This IR product is readily available near the location where I reside 5. I can handle any (such as money, time, information) difficulties associated with my buying decision	[117]
**Purchase** **Intention (PI)**	1. I will consider buying an IR product 2. I would be willing to recommend IR products to my colleagues, friends, relatives, or families 3. I will buy an IR product shortly 4. It will take me a long time to buy IR products	[118, 119]

Source: Tai [11]

## Results

### Reliability analysis

Reliability can generally be divided into split-half reliability, replica reliability, test–retest reliability, and internal consistency reliability. Internal consistency reliability is used to measure reliability and split-half of the executive functioning for score differences and merit replication [120–122]. Thus, the degree of internal consistency can be reproduced across diverse groups within populations because the reliability test creates high-reliability consistency. Cronbach's α is the internal consistency reliability index that is adopted in most studies and this methodology was used in this study [122, 123]. The Cronbach’s α value is a measure of the reliability of the questions where the responses are based on a Likert scale. To consider the content of a questionnaire reliable, the Cronbach α values must meet a minimum threshold of 0.7 or higher [120, 124]. The Cronbach’s α values for EU, PP, (BP), A, SN, PBC were all above 0.7 (Table 2). As such, the questions asked in the questionnaire were deemed to be reliable.

**Table 2:** Summary of the Cronbach’s α reliability analyses for the overall shopper group

Variables and TPB Constructs	Number of Questions	Question Sources	Cronbach’s α
Ease of Usage (EU)	7	[83, 106–110]	0.851
Product Price (PP)	3	[111, 112]	0.777
Brand Perception (BP)	7	[113]	0.832
Attitudes (A)	5	[114]	0.828
Subjective Norms (SN)	4	[115, 116]	0.824
Perceived Behaviour Control (PBC)	5	[117]	0.798
Purchase Intentions (PI)	4	[118, 119]	0.775

The Cronbach’s α values for the online shopper group are by definition included in the overall shopper group. source: Tai [11]

### SEM outcomes

To verify the causal relationship between the TPB constructs and variables in this study a two-stage SEM analysis was performed following Anderson and Gerbing [125] and Williams and Hazer [126]. The first stage was focused on each variable and a Cronbach’s α coefficient analysis was performed on those constructs. A stable measurement model was developed through an analysis of reliability, convergent validity, and discriminant validity. The advantage of using the two-stage method is that the influence of the measurement and structural modes can be separated. If the reason for the insignificance of the test results cannot be determined, then the influence of the reliability and validity of the measurement model, or problems among the constructs in the structural model can be determined.

**Fig. 2:**
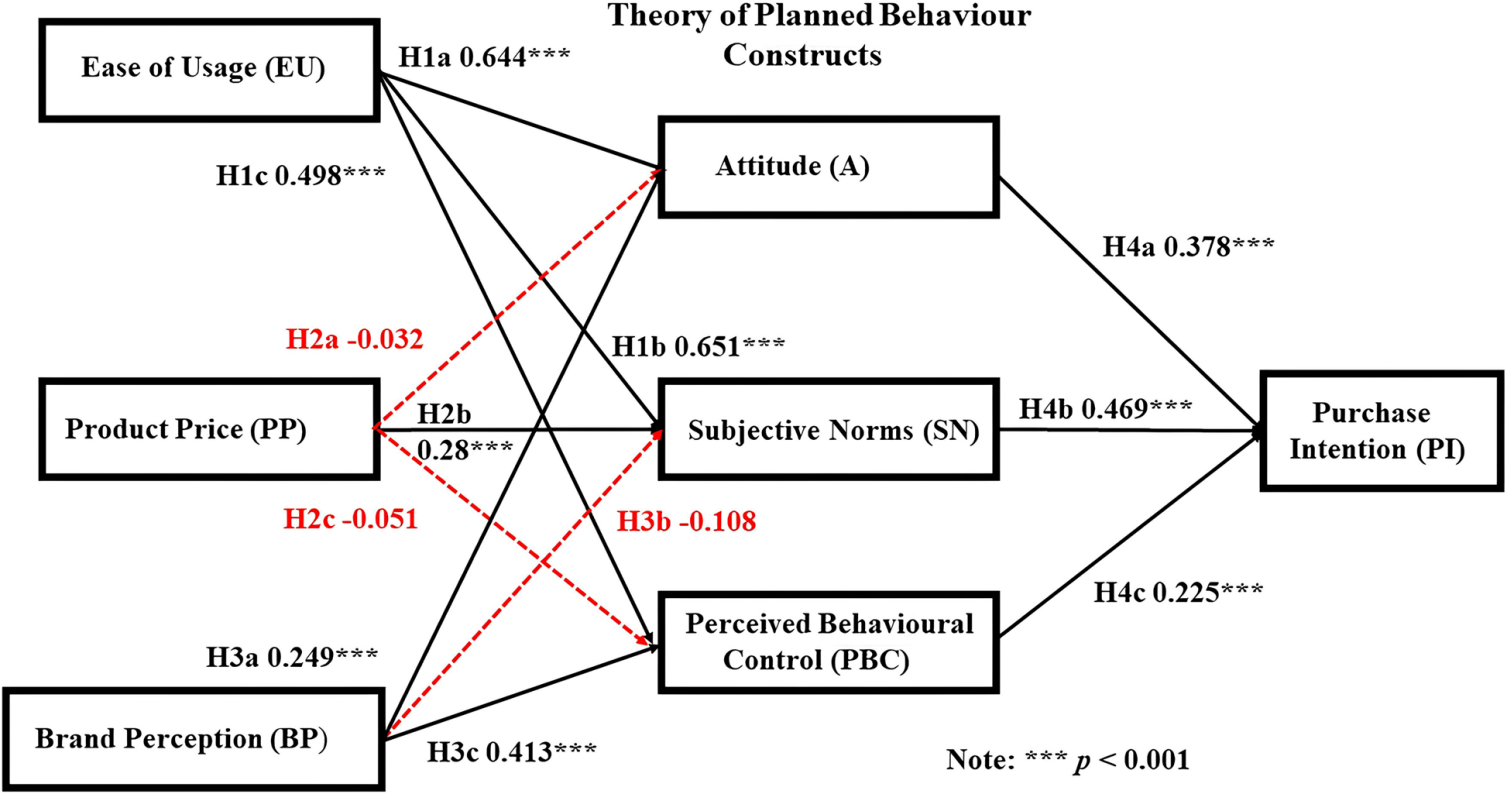
Structural Path Coefficients and the causal impacts of the variables on the TPB Constructs and PI for the Overall Shopper group. Source: Tai [11]

The test results for the Overall Shopper group supported H1a, H1b, and H1c (EU ➔ A, SN, and PBC), H2b (PP ➔ SN), and H3a and H3c (BP ➔ A and PBC), and H4a, H4b, and (A, SN, and PBC ➔ PI). Hypotheses that were not supported and therefore rejected included: H2a (PP ➔ A), H2c (PP ➔ PBC), and H3c (BP ➔ SN) (Fig. 2; Table 3).

**Table 3:** Structural Path coefficients and impacts of the variables on the TPB constructs and PI of the online and offline shopper groups. source Tai [11]

Structural Path Analyses	Hypotheses	Online Shopper Structural Path Coefficient Values	Online Shopper Hypothesis Results	Hypotheses	Overall Shopper Structural Path Coefficient Values	Overall Shopper Hypothesis Results
Ease of Usage → Attitude	H1a	0.838	Supported	β1a	0.644	Supported
Ease of Usage → Subjective Norms	H1b	0.651	Supported	β1b	0.651	Supported
Ease of Usage → Perceived Behavioral Control	H1c	0.79	Supported	β1c	0.498	Supported
Product Price → Attitude	H2a	(-0.127)	**Not Supported**	β2a	(-0.032)	**Not Supported**
Product Price → Subjective Norms	H2b	0.28	Supported	β2b	0.28	Supported
Product Price → Perceived Behavioral Control	H2c	(-0.155)	**Not Supported**	β2c	0.051	**Not Supported**
Brand Perception → Attitude	H3a	0.142	**Not Supported**	β3a	0.249	Supported
Brand Perception → Subjective Norms	H3b	0.005	**Not Supported**	β3a	0.108	**Not Supported**
Brand Perception → Perceived Behavioral Control	H3c	0.133	**Not Supported**	β3c	0.413	Supported
Attitude → Purchase Intention	H4a	0.105	**Not Supported**	β4a	0.378	Supported
Subjective Norms → Purchase Intention	H4b	0.753	Supported	β4b	0.469	Supported
Perceived Behavioral Control →	H4c	0.176	**Not Supported**	β4c	0.225	Supported

### Reliability measurement indices

The reliability measurement indices of the Overall Shopper group showed positive relationships between EU ➔ A (H1a = 0.644***, *p* < 0.001), EU ➔ SN (H1b = 0.651***, *p* < 0.001), EU ➔ PBC (H1c = 0.498***, *p* < 0.001). As such, H1a, H1b, and H1c are strongly supported (Fig. 2; Table 3). The relationships between PP ➔ A and PBC do not have any significant effects on these TPB constructs, so H2a and H2c are rejected. The only the positive relationship seen is between PP ➔ SN (H2b = 0.28***, *p* < 0.001), therefore H2b is supported. Positive relationships are seen between BP ➔ A (H3a = 0.249***, *p* < 0.001) and BP ➔ PBC (H3c = 0.413***, *p* < 0.001) and H3a and H3c are supported. The relationship between BP ➔ SN is not supported and therefore is rejected. The relationships between A ➔ PI (H4a = 0.378***, *p* < 0.001), SN ➔PI (H4b = 0.469***, *p* < 0.001), and PBC ➔ PI (H4c = 0.225***, *p* < 0.001) are positive and H4a, H4b, and H4c are supported.

**Fig. 3:**
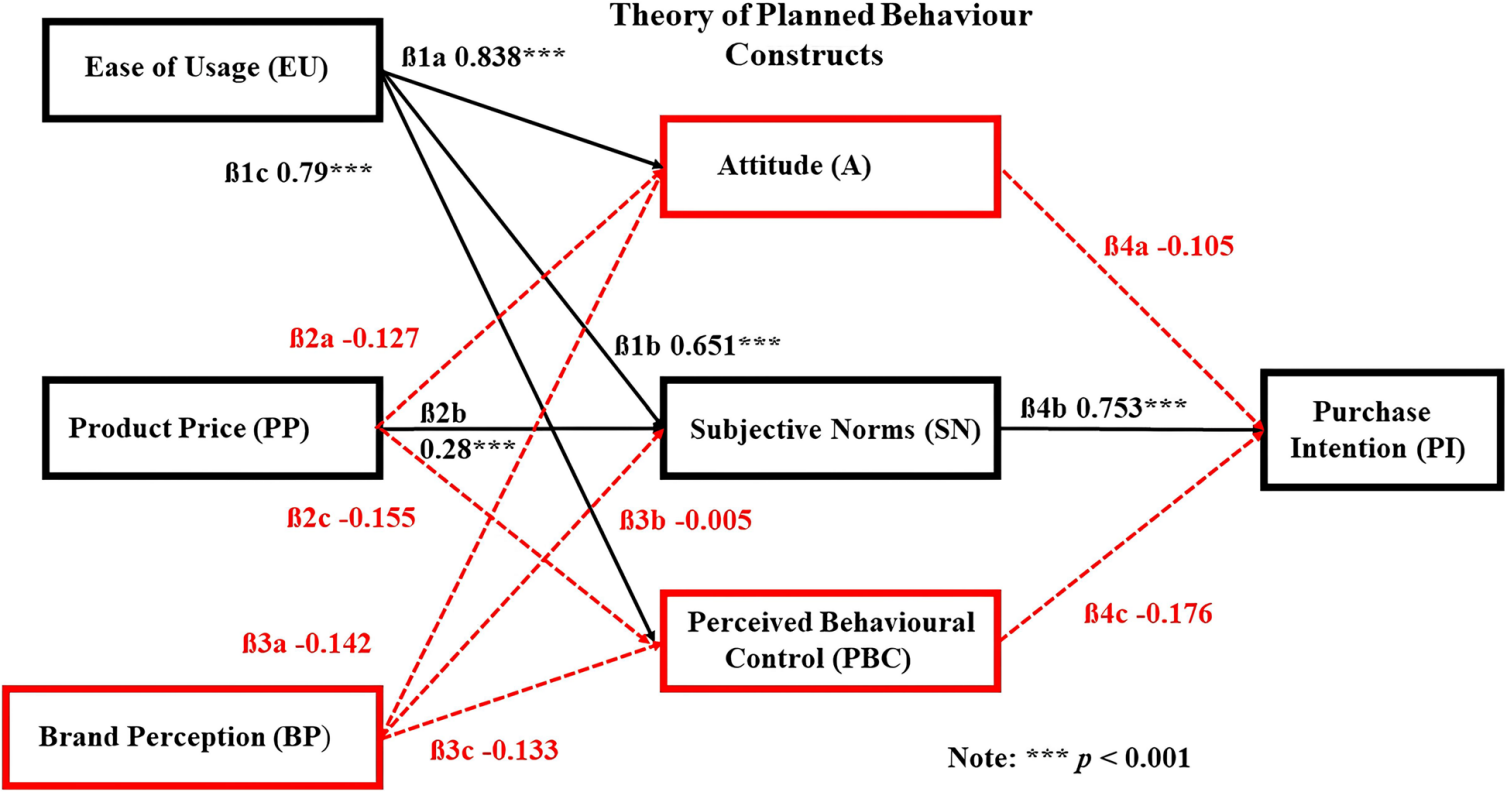
Structural Path Coefficients and the causal impacts of the variables on the TPB Constructs and PI for the Online Shopper group. Source: Tai [11]

The reliability measurement indices of the Online Shopper group showed positive relationships between EU ➔ A (β1a = 0.838***, *p* < 0.001), EU ➔ SN (β1b = 0.651***, *p* < 0.001), EU ➔ PBC (β1c = 0.79***, *p* < 0.001). As such, β1a, β1b, and β1c are strongly supported (Fig. 3; Table 3). The relationships between PP ➔ A and PBC do not have any significant effects on these TPB constructs, so β2a and β2c are rejected, but the relationship between BP ➔ SN is positive (β2b = 0.28***, *p* < 0.001), and β2b is supported. There are no positive relationships between BP ➔ A, BP ➔ SN, or BP ➔ TPB so β3a, β3b, and β3c are rejected. The only TPB construct that positively influences PI is SN (β4b = 0.753***, *p* < 0.001), so β4b is supported, while β4a and β4c are rejected.

To evaluate the fit of the model (Fig. 2) and observed data (Fig. 3; Table 3) three measurement indicators and the suggestions and opinions in Hair et al. [127] were used. The measurement indicators included: (a) Absolute Fit; (b) Incremental Fit; and (c) Parsimonious Fit.

- Absolute Fit Measures: These measures are used to determine the degree to which the model based on the Overall Shopper group data can predict the covariance or correlation matrix by evaluating the fit between the proposed model and actual data. The measurement indicators include: Chi-Square value, Ratio of Chi-Square value to the degree of freedom, Goodness of Fit Index, average Residual Mean Square Root, Root Mean Square Error of Approximation, and Adjusted Goodness of Fix Index (Table 4). The absolute fit index values of our model were: χ2 = 671.785, df = 401, χ2/df = 1.675, GFI = 0.829, AGFI = 0.788, and RMSEA = 0.058. The χ2 value is significant and many have pointed out that the χ2 value is sensitive to sample size when the number of samples exceeds 200 [128–132]. Therefore, despite having an acceptable model with a significant χ2 value and test results supporting the null hypotheses, there is no guarantee that the obtained model is the best. Therefore, we advocate using other fit statistics when using AMOS. The results of these subsequent analyses can then be used to determine the fitness of the model. Although the Normed Fit Index (NFI) and AGFI values did not reach their respective standards, they were still within their acceptable range standard. The other indicators all met their respective standards (Table 4).
- Incremental Fit Measure: This measure is a comparison of fit between target and null models. This class of fit indices quantify the proportional improvement in the fit of a target model relative to a null model [133]. Based on the statistical criteria employed in this study, the incremental fit measures were instructive and complemented the other fit method results because they not sensitive to the small or moderating effects of a large sample size, as opposed to the manner other causal models are evaluated [134, 135]. They are reported together with other descriptive statistics that help understand the results better. The measurement indicators of this test included the NFI, which was 0.828 and the Comparative Fit Index, which was 0.922 and both were within their acceptable ranges (Table 4).
- Parsimonious Fit Measures: These measures adjust the fit measurement so that the different estimated coefficients can be compared to one another to determine the degree of suitability that can be obtained for each estimated coefficient. This is to say that when the AMOS results are expected to reach a certain level of suitability, the number of parameters should be estimated in the causal model when the measurement index includes a Parsimony Normed Fit Index and Parsimony Goodness of Fit Index. The parsimony fit indices for the model in this study were PNFI = 0.714 and PGFI = 0.67. Both reached an acceptable range of > 0.500 and based on the indicators used, the model fit of our study using AMOS is acceptable (Table 4).

**Table 4:** Summary of the model fit for the overall shopper group

Data Fit Measures	Measurement Indicators	Standard Values	Values derived in this study	Assessment Results
**(a) Absolute** **Fit Measures**	χ^2^	*P* > α = 0.05	671.785***	**Not Standard**
	χ^2^: df	< 3 or 5	1.675	Standard
	GFI	> 0.9 or 0.8	0.829	Standard
	AGFI	> 0.9 or 0.8	0.788	**Near Standard**
	RMSEA	< 0.08 or 0.1	0.058	Standard
**(b) Incremental** **Fit Measures**	NFI	> 0.9	0.828	**Near Standard**
	IFI	> 0.9	0.923	Standard
	TLI / NNFI	> 0.9	0.909	Standard
	CFI	> 0.9	0.922	Standard
**(c) Parsimonious** **Fit Measures**	PNFI	> 0.5	0.714	Standard
	PGFI	> 0.5	0.67	Standard

Chi square (χ^2^), (*DF*) degrees of freedom, (*GFI*) Goodness of Fit Index, (*AGFI*) Adjusted Goodness of Fix Index, (*RMSEA*) Root Mean Square Error of Approximation, (*NFI*) Normed Fit Index, (*IFI*) Incremental Fit Index, (*TLI*) Tucker Lewis Index, (*NNFI*) Non-Normed Fit Index, (*CFI*) Comparative Fit Index, (*PNFI*) Parsimony Normed Fit Index, (*PGFI*) Parsimony Goodness of Fit Index. The Model fit for the Online Shopper group are by default included in the Overall Shopper group. Source: Tai [11]

***P ≤ 0.001

The variables that were measured included EU, PP, BP, AT, SN, PBC, and PI and all reached their respective standard (Table 4). Therefore, the fit of the internal structure of the research model is good.

### Comparison between the overall and online shopper groups

**Fig. 4:**
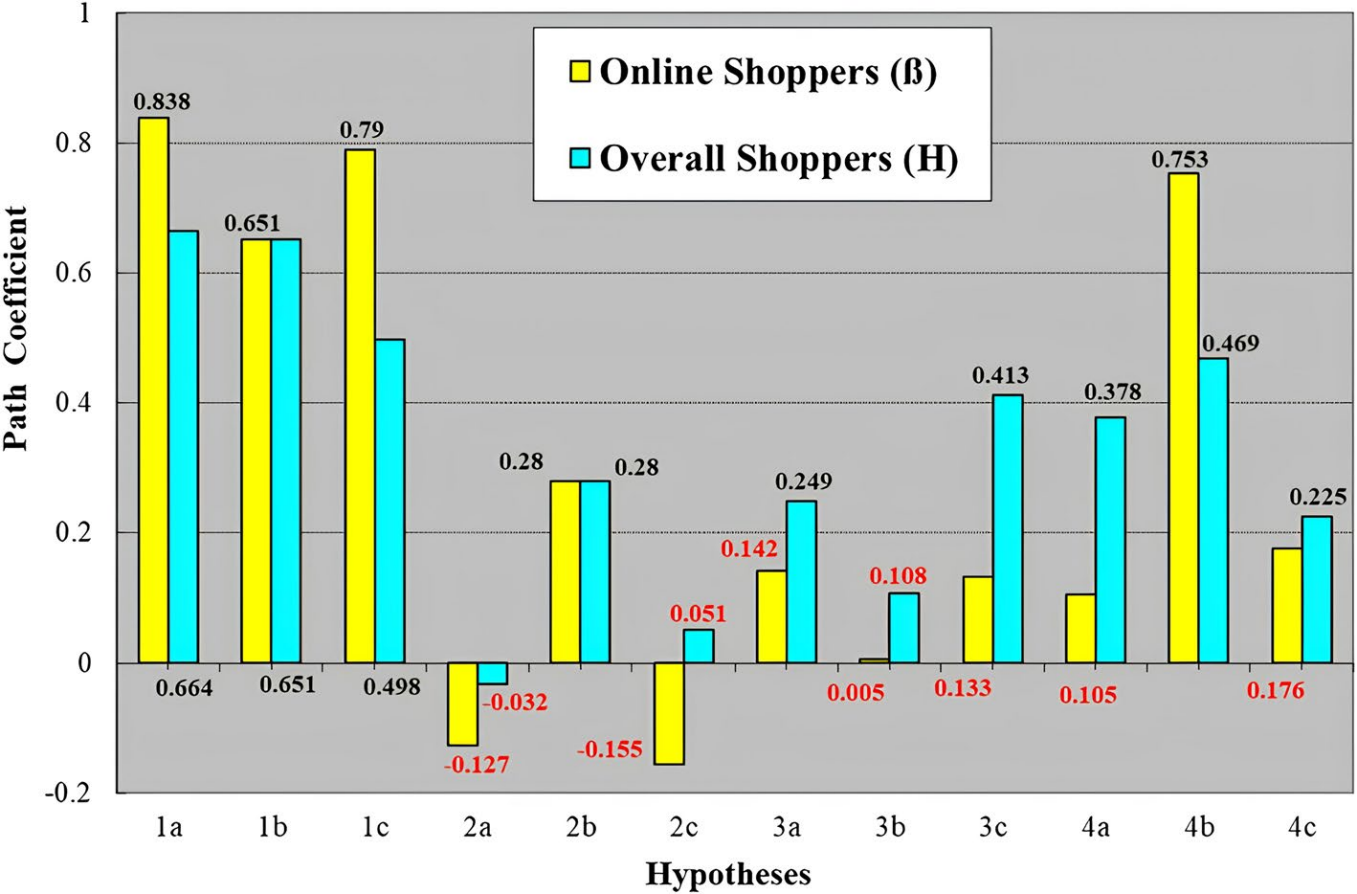
Bar chart showing the structural path coefficient values and relationships between the online and overall shopper groups for each hypothesis. Source: Tai [11]

To understand better the impact of EU, BP, and PP of the Online Shopper group on the TPB constructs and PI for consumer purchases of IR goods and services we first performed the analyses on the Overall Shopper group. This provided a baseline of the causal impacts on the TPB constructs and ultimately the impact of the impact of the TPB constructs on consumer PI for IR goods and services for the entire population. The Overall Shopper group was then segregated into two groups based on shopping preferences, Online or Offline. Specific shopping behaviour variables between the two groups could be compared with one another as a way to determine the variables that influenced the TPB of each group and impact to these consumers to try and/or buy IR goods and services. As noted previously, future contributions will be used to examine Offline-Online Shopper group, Offline-Overall Shopper group, Online-Offline-Overall Shopper group, and the impact of demographic variables on the TPB relationships examined in this study. An SEM analysis was performed on the Overall (Fig. 2) and Online Shopper (Fig. 3) groups and the results were compared and interpreted (Table 4). To better visualize the effect of splitting the Online Shopper from the Overall Shopper group, a bar chart illustrating the strengths of the structural path coefficient relationships between the two groups are presented (Fig. 4).

Hypothesis 1 The hypotheses H1a/ß1a, EU➔ A; H1b/ß1b EU ➔ SN, and H1c/ß1c EU ➔ PBC for both groups are positive and accepted. However, the strength of the structural path coefficients between EU ➔ A and EU ➔ PBC in the Online Shopper group are stronger than the Overall Shopper group, while the strength of the EU ➔ SN structural path coefficient is the same in both groups. Hypothesis 2: The relationships between PP ➔ A and PP ➔ PBC in both groups are rejected, but the relationship in both groups between PP ➔ SN, is positive, so hypothesis H2b/ß2b is supported. Even though H2a/ß2a and H2c/ 2c are rejected the strengths of the structural path coefficient relationships between PP ➔ A and PP ➔ PCB are stronger in the Online Shopper group compared to the Overall Shopper group. The structural path coefficient strength for H2b/ß2b, PP ➔ SN is the same for both groups. Hypothesis 3: In the Online Shopper group, the relationships between BP ➔ A, BP ➔ SN, and BP ➔ PBC are not supported and hypotheses ß3a, ß3b, and ß3c are rejected. The BP ➔ A and BP ➔ PBC relationships are positive in the Overall Shopper group and H3a and H3c are supported, but the relationship between BP ➔ SN is not supported and hypothesis H3b is rejected. The strength of the structural path coefficient relationships seen in H3a, H3b, and H3c of the Overall Shopper group is considerably stronger than the Online Shopper group. Hypothesis 4: The only the positive relationship in the Online Shopper group that is seen in H4 is between SN ➔ PI and hypothesis ß4b is supported, while the relationships between A ➔ PI and PBC ➔ PI are not supported and hypotheses ß4a and ß4c are rejected. The strengths of the structural path coefficients for H4a and ßH4c are stronger for the Overall Shopper group compared to the Online Shopper group, while ß4b is stronger for the Online Shopper group compared to the Overall Shopper group (Fig. 4).

## Discussion

The results of our analyses indicate important similarities differences between the Overall and Online Shopper groups with respect to the PI of IR goods and services even though the Online Shopper group is a subset of the Overall Shopper group (Figs. 2, 3, 4; Table 3). Consumer responses from both populations indicate the Ease of Usage (EU) for IR goods and services is an important factor that positively impacts their A, SN, and PBC, which in turn positively influences their PI for IR goods and services. Results of this analysis show consumers that often shop online don’t care about the brand of the product. As long as the product functions for the purpose it was designed and EU is not difficult, the consumer will subjectively want to purchase the goods or services. From a psychological point of view, this is one of the reasons why we often make impulsive purchases [136, 137]. The appearance and description of the products available online, coupled with innovative marketing techniques can easily sway or change consumer opinions on purchases. Huang [47] indicated that the Perceived Ease of Usage and Perceived Usefulness for health-intelligent products influenced consumer PI, which supports our results. Sun et al. [48] showed that the effects of A, SN, and PBC impacted the functionality and utility of mobile payments on consumer repurchasing intentions, while Rachmawati et al. [49] and Rini et al. [50] demonstrated the significant impact of the perceived benefits of EU, SN, and personal innovation on A for using social media, and the variables associated with online shopping behaviour intentions to purchase goods and services. The results of these studies further support those of this study.

With respect to Brand Perception (BP), the Overall Shopper group favored obtaining their IR-related goods and services at brick-and-mortar stores compared to the Online Shopper group. The ability to see and/or handle products, compare prices to determine the best value, interact with humans and ask questions, and possibly even socialize is a preferred shopping method compared to the Online Shopper group [138]. The Overall Shoppers evaluated their A and PBC capabilities, which then generated their PI. The results of this study indicate the PBC did not affect the PI of the Online Shoppers, but it did for Overall Shopper group. The same result applies to A, which had no positive effect on the PI of the Online Shopper group for IR goods and services, but it did for Overall Shopper group. The A of Online and Overall Shoppers may vary depending on several factors, including target audience, product, and marketing strategies, which could impact or influence their PI for IR goods and services.

Attitude is the evaluation of a product or service, which then impacts consumer perceptions of the worth and the value of the product. Social Norms, personal values and norms, and situation then impact consumer intentions to purchase IR goods and services. In a study of Online and Offline shoppers Brüggemann and Pauwels [139] examined consumer attitudes and purchasing behavior and found that the Online Shoppers had a higher preference for national brands and were less conscious about product price compared to the Offline Shoppers. The results for the Online Shopper group of this study are consistent with their results. The relationship between the Online and Overall Shopper group EU on A for IR goods and services impacts their PI. This finding is supported by others that utilized the TPB for Online and Offline Shopper behaviours [47–50, 140].

Product Price had no significant effect on A and PBC in either group, but had a positive effect on their SN, which then had a positive effect on their respective PI. The relationships between PP, A, and PBC are not supported by the Online or Overall Shopper group results. Shopper perceptions of product usefulness will vary with community demographics, location, culture, market demand, season, and the questions asked, which then contribute to PP fluctuations. Huang [47] indicated PP is closely linked to Online Shopper perceptions. The Online Shopper group have positive and statistically significant support for PP, which is influenced by their respective SNs that then influence their PI for goods and services. The results of previous studies show PP influences A to buy products believed to be low in price based on assurances from stores and equality in price outcomes [58, 141, 142]. Customer perception of price fairness is also a key factor that affects fulfilment and customer loyalty to the store and product [143].

## Conclusions

The results of this study are interesting because they demonstrate that minor/subtle changes, such as how a group is defined can have how target groups are defined, which then impacts our understanding of a group’s behaviour. This then can have significant impacts on correctly/incorrectly targeting groups and developing marketing strategies for goods and services for target groups. Furthermore, it illustrates the importance of designing studies, survey questions, data management, and analyses that are then used as a basis for advertising and marketing campaigns. Trends or relationships can be lost or minimized when the data are pooled and become averaged.

Subjective norms had a positive effect on PI in all groups, while A and PBC had a partial effect on PI. Thus, the recommendations for this study indicate IR cannot completely replace professional worker positions and suggestions are provided for today's high-tech industry and e-commerce. Special personnel are still needed to maintain services, retain professional knowledge, and brick-and-mortar stores cannot be eliminated. Nonetheless, IR is likely to replace low-end and routine jobs such as store service personnel, factory operators, medical service personnel, and family companions. Therefore, within the supply chain, constructs such as PP and product cost, brick and mortar stores and sales personnel will likely be reduced, while shopping online will continue to increase. Through such commercial operations, consumers can more easily obtain information, news, and product use, to make better comparisons and decisions. Consumers are increasingly becoming more dependent on Internet services and many high-tech industries are gradually moving towards the convenience of Big Data and Cloud Computing, which will be the essence of commercial services.

The results related to BP are intriguing (Fig. 4). The Overall Shopper group shows the relationships between BP ➔ A and BP ➔ PBC are positive, but the Online Shopper group structural path coefficients were rejected. The structural path coefficient values show a 57%, 463% and 32% decrease between the BP ➔A, BP ➔SN, and BP ➔PBC for the Overall and Online Shopper groups. Although the BP ➔SN relationship for H3 is negative for the Overall Shopper group, the value compared to the Online Shopper group is much stronger (-0.108 versus -0.005). The substantial drop in the BP ➔SN relationship suggests shoppers may no longer be concerned as much about conforming to SN compared to the past. These results may be due to variables such as consumer age and the amount of time consumers are spending shopping at their computers and smartphones and it would be inappropriate to speculate until these variables are tested. Clearly a more detailed examination of these trends that are focused on specific subsets of the population is warranted.

## Opportunities

**Fig. 5:**
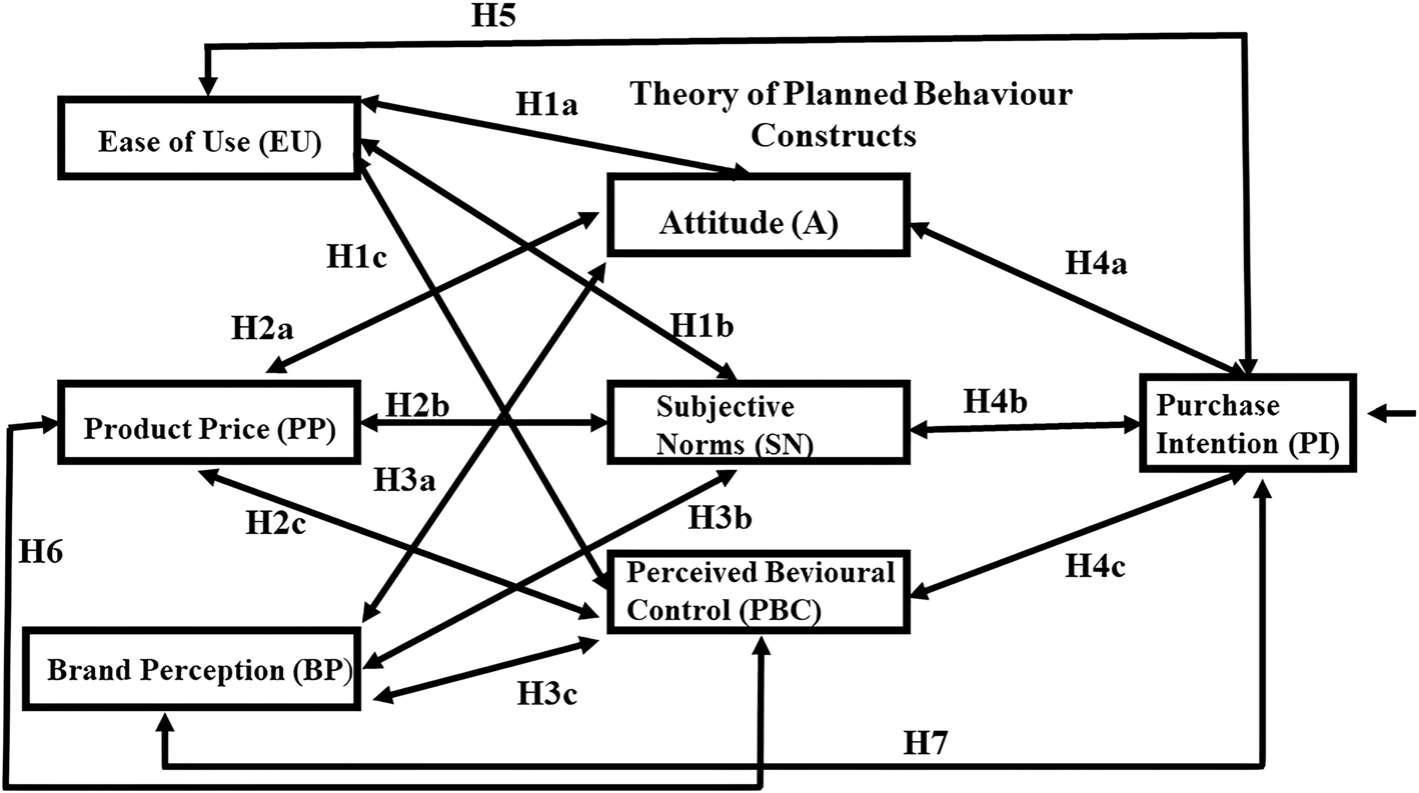
Opportunities for further research. Source: Tai [11]

A model for further research on the constructs were tested that were associated with the PI of IR goods and services for the Online Shopper group is provided (Fig. 5). We hypothesize that the EU will be positively related to PI (H5), PP will be positively related to PBC (H6), and BP will be positively related to PI (H7). Product Price is a crucial factor that affects a consumer's decision to buy IR or any goods and services. Everyone has a budget or maximum price that they are willing to pay for IR product or service. Essentially, price and the perception of price greatly affect a consumer's decision to purchase a product. It has been hypothesized that the Perceived Ease of Usage affects Perceived Usage and PI, which has been studied in terms of why a consumer purchases a particular brand. Purchase Intention has been identified as the consequence of various factors, such as A, which is a predictor of satisfaction. However, self-efficacy plays an important role in Information System acceptance, electronic services, and Web-based Information Systems.

Understanding these relationships better may assist in regulating the behaviour of IR purchases such as motivation, persistence, endurance, and diligence, to overcome possible difficulties. Brand Perception should be studied extensively to understand its relationship with the EU and PP. Aspects that also require further consideration that were not assessed due to space limitations were the impacts of the demographic variables associated with Online and Offline purchases of IR goods and services by consumers. As noted by Brüggemann and Pauwels [139], aspects such as age, attitude, purchase shares for fair trade, products differences between Online and smartphone purchases are recognized. In addition, Online to Offline (O2O) commerce multi-channel management, and the extent of smartphone versus desktop/laptop use to acquire goods and services are emerging concepts that need further understanding, especially for businesses to be successful in the future [144].

In these types of studies, the presumption is that positive or negative relationships in a model are unidirectional. It would be of interest to know whether these presumptions are un- and/or bi-directional, and the possibly that bidirectional relationships are present, but missed because we’ve assumed the relationships are always one-way. Certainly, a much deeper and more comprehensive analyses of additional variables is warranted and while many of these analyses are already being performed, there are substantial opportunities to build on this work.

## Data Availability

Data is provided within the manuscript and available upon request by contacting YHT.
